# Nutritional attitudes of professional women volleyball players: a cross-sectional study from Türkiye

**DOI:** 10.3389/fnut.2025.1579953

**Published:** 2025-07-17

**Authors:** Oktay Sahin, Vesile Sahiner Guler, Mehmet Behzat Turan, Keziban Yoka, Osman Yoka, Yahya Polat

**Affiliations:** ^1^Department of Physical Education and Sports, Faculty of Sports Sciences, Gümüşhane University, Gümüşhane, Türkiye; ^2^Department of Physical Education and Sports, Institute of Health Sciences, Erciyes University, Kayseri, Türkiye; ^3^Department of Physical Education and Sports, Faculty of Sports Sciences, Erciyes University, Kayseri, Türkiye; ^4^Department of Physical Education and Sports, Institute of Social Sciences, Nigde Omer Halisdemir University, Nigde, Türkiye

**Keywords:** sports nutrition, nutrition knowledge, female elite athletes, dietary habits, sociodemographic factors, nutrition education

## Abstract

**Background:**

Nutritional habits are a critical factor influencing the performance and recovery of professional athletes. Understanding how demographic variables affect nutrition-related attitudes is essential for developing targeted interventions.

**Objective:**

This study examined the nutritional attitudes of professional female volleyball players in Türkiye based on various demographic characteristics.

**Methods:**

The study included 386 licensed female volleyball players from the Turkish Women's Volleyball Super League, First League, and Second League. Data were collected using a demographic questionnaire and the Attitude Scale for Healthy Nutrition. Normality was assessed using the Kolmogorov-Smirnov test and skewness-kurtosis values. Statistical analyses included *t*-tests, ANOVA, LSD *post-hoc* tests, and partial correlation.

**Results:**

Significant differences in nutritional attitudes were found based on league level, smoking status, years of experience, and income level (*p* < 0.01). No significant differences were observed based on education level. Among the sub-dimensions, the malnutrition component showed no significant differences across most variables. Super League athletes scored significantly higher in nutritional knowledge and positive nutritional behaviors.

**Conclusion:**

The nutritional attitudes of professional volleyball players vary considerably according to key demographic factors. These findings highlight the importance of targeted nutrition education, particularly for lower-league athletes, to enhance performance and reduce disparities in nutritional knowledge.

## 1 Introduction

Volleyball is a sport that demands maximum endurance (respiratory functions, anaerobic and aerobic capacity), strategy, reaction time, balance, agility, speed, flexibility, strength, athletic efficiency, and control (1). Due to their engagement in intensive training programs and frequent competitions, professional athletes differ significantly from the general population. Athletic performance is influenced by physical training and genetic predisposition, training time, and the athlete's physical, psychological, and physiological characteristics, including nutrition (2). Achieving optimal athletic performance requires an integrated approach in which training and nutrition work synergistically (3). Nutrition is fundamental in supporting an athlete's training and overall performance. For adult athletes, maintaining a balance between caloric intake and energy expenditure is essential for performance, recovery, and long-term health. For young athletes, nutrition must support ongoing growth and development alongside training demands (4). However, athletes often face challenges in achieving optimal dietary intake due to harmful nutritional practices and various limiting factors. These include time constraints, financial limitations, social and demographic influences, lack of cooking skills and equipment, insufficient institutional support, cultural preferences, and inadequate nutrition knowledge (5). The consequences of poor nutrition in professional athletes can be severe. Insufficient energy intake may hinder the body's adaptation to physical activity, decreasing lean body mass, muscle strength, and endurance. It can also impair immune function and increase the risk of injuries and other health complications (6). Athletes may be particularly vulnerable to unhealthy eating behaviors due to the intense pressure and competitive nature of sports, which can contribute to disordered eating patterns (7–9). A comprehensive understanding of food and nutrition empowers athletes to manage their dietary needs effectively. Knowledge about food, such as portion sizes, nutritional content, and appropriate meal timing, helps individuals make informed choices based on their unique requirements (10). Nutritional counseling can further enhance athletes' dietary practices by expanding their understanding and helping them tailor their intake to their performance goals (11). Adequate nutrition has been consistently identified as a key factor in achieving peak performance, promoting post-exercise recovery, and reducing the risk of sports-related injuries (12). Adopting sports nutrition guidelines significantly influences athletes' nutrient intake and is increasingly recognized for its performance-enhancing effects (13). However, studies suggest that many athletes lack sufficient nutritional knowledge, often leading to poor dietary choices. While understanding nutritional principles is vital, motivating athletes and raising awareness about proper nutrition are equally crucial to ensure adherence to optimal nutritional practices (14).

**Figure 1:**
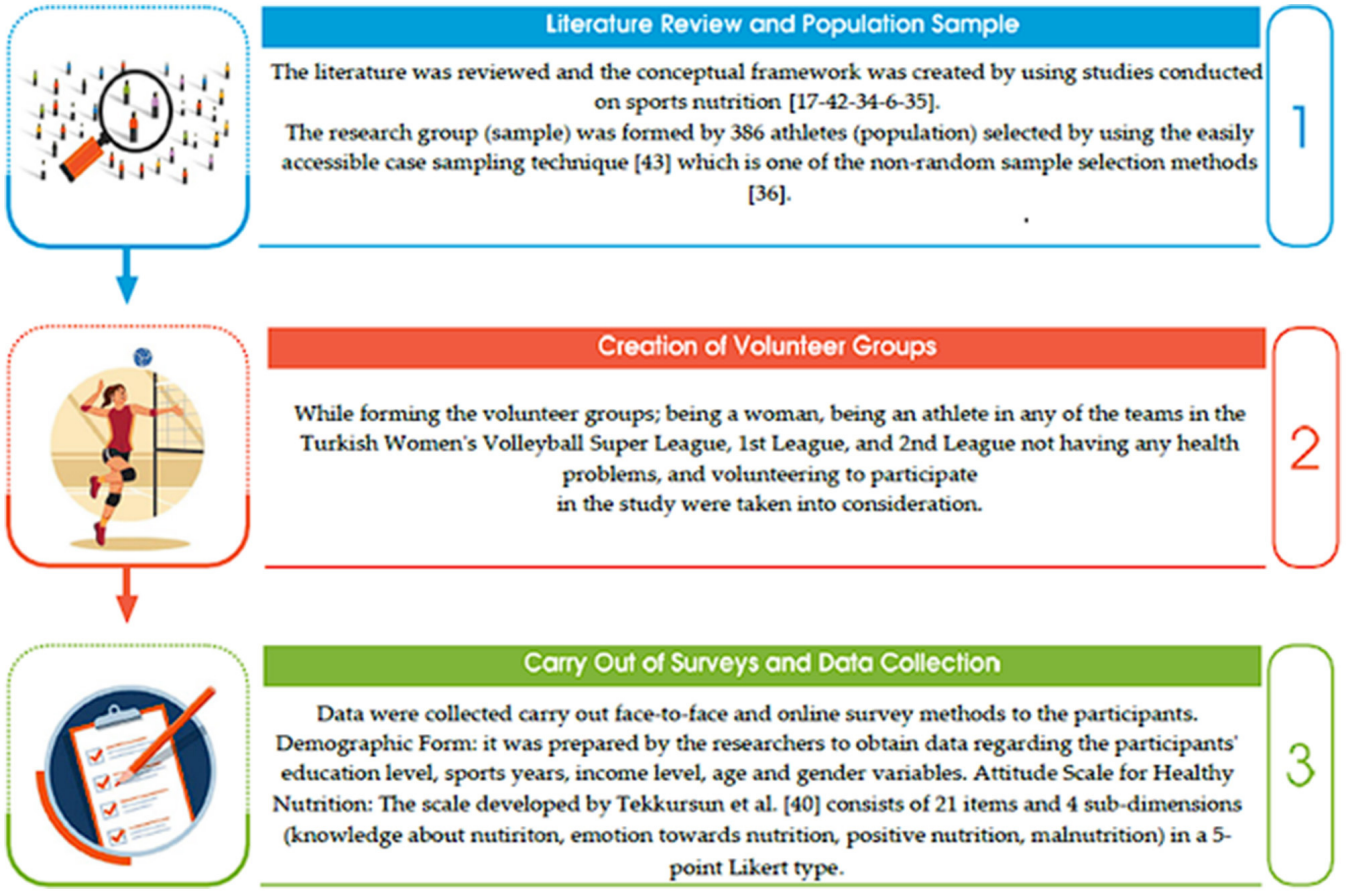
Flowchart.

A literature review shows that while several studies have used the Attitude Scale for Healthy Nutrition, their focus and sample characteristics differ from the current study. Adatepe and Çelik (15) applied the scale to mixed-gender amateur football players, and Bidil (16) to younger badminton players. Although similar in methodology, these differ in terms of sport and demographics. Other research, such as Holden et al. (17) on Paralympic volleyball players, Gümüşdag and Kartal (18) on handball players, and Spronk et al. (19) on elite team athletes, focused on nutritional knowledge rather than attitudes, using different tools. Hepyüksel Taşkiran (20) examined nutrition habits during the pandemic across various sports, while Cockburn et al. (21), Yahia et al. (22), and Andrews et al. (23) involved non-athlete or mixed groups with different instruments. Overall, studies targeting female volleyball players using the Attitude Scale for Healthy Nutrition are scarce. This study addresses that gap, comparing its sample and tools to Chowela et al. (24), who studied NCAA Division I baseball players. While both examined nutrition and used similar tools, they differ in sample type, scope, and performance measures. The present research of these studies focuses specifically on professional female volleyball players competing in Türkiye's top three leagues. These athletes are widely recognized and appreciated by society and serve as role models, particularly in light of the growing popularity of women's volleyball in Türkiye and the recent international successes of the national women's team. This study includes professional athletes who comprise 31.22% of the total population of interest, thereby enhancing the generalizability and reliability of the findings. In contrast to previous research, this study demonstrates that various demographic characteristics significantly influence athletes' nutritional attitudes and behaviors. These findings align with the well-established notion that nutrition impacts athletic performance. Moreover, the observed differences are influenced not only by physical conditions but also by social factors. Even among athletes with 4 or more years of professional experience, demographic variables continue to affect nutritional behavior, highlighting the ongoing need for nutritional education at both amateur and professional levels. Research on sports nutrition is expanding rapidly, contributing to the literature by strengthening the evidence base and proposing new approaches. Many international organizations recognize sports nutrition as an evolving scientific discipline with a dynamic structure and are actively researching this area (25, 26). Based on this context, the present study aims to examine the attitudes and knowledge levels regarding nutrition among professional female volleyball players competing in the Turkish Super League, First League, and Second League, with attention to various demographic variables. Given these athletes' elite status and demanding training schedules, they are typically difficult to access for research purposes. This sampling ratio enhances the external validity and representativeness of the findings. While the existing literature generally focuses on amateur athletes or mixed samples from various sports disciplines, this study distinguishes itself by examining professional female volleyball players and utilizing a large sample size. Furthermore, by considering the effects of demographic factors such as education level and league level on nutritional attitudes, even among professional athletes, this study provides innovative data to support the development of individualized nutrition programs and athlete support services. The sustainability of athletic performance is not solely dependent on physical training; it is also closely linked to a science-based approach to nutrition. Notably, the findings indicate that nutritional attitudes among professional female volleyball players can vary significantly depending on demographic variables. This highlights the urgent need to move away from generic, “one-size-fits-all” nutrition strategies and toward individualized, sport-specific approaches in sports nutrition education. Applied, sustainable, and expert-supported programs currently lacking in the field should aim to deliver information and facilitate meaningful behavioral change in athletes. With its large and professionally engaged sample, this study illuminates this need and addresses a notable gap in the literature. The flow chart of the study is given in Figure 1.

The objectives of this study are as follows:

To determine the differences in nutritional attitudes based on participants' smoking status.

To determine the differences in dietary attitudes based on participants' income levels.

To determine the differences in nutritional attitudes based on participants' years of sports experience.

To determine the differences in nutritional attitudes based on participants' league level.

To determine the differences in dietary attitudes based on participants' education level.

To examine whether nutritional attitude differences persist across variables when controlling for league level.

## 2 Materials and methods

### 2.1 Participants

An a priori power analysis was conducted using *G*Power 3.1^*^ to determine the required sample size for a one-way omnibus ANOVA with fixed effects. The study assumed a medium effect size (*f* = 0.25), a significance level of α = 0.05, and a desired power of 1–β = 0.95. Four groups were specified for comparison. Under these parameters, the analysis indicated a required total sample size of 280 participants. The corresponding non-centrality parameter (λ) was 17.50, with a critical *F* value of 2.6373. The degrees of freedom were df1 = 3 (numerator) and df_2_ = 276 (denominator). The achieved power with *N* = 280 was calculated as 0.9510, confirming that this sample size could detect medium-sized group differences at the specified alpha and power levels (27).

Participants were selected using a simple random sampling method (28). The inclusion criteria for participants were as follows:

Being 18 years of age or older.

Actively competing in the Turkish Women's Volleyball Super League, First League, or Second League.

Having been a licensed athlete in one of these leagues for at least 4 years.

The questionnaire was distributed electronically to participating clubs via a secure online platform. Athletes who met the predefined inclusion criteria and provided *informed, voluntary consent* completed the scale.

As a result of this sampling procedure, 386 professional volleyball players were included in the study. When considering the total population and referring to sample size tables based on population size and acceptable sampling error levels (0.01, 0.02, 0.03, 0.04, and 0.05), it can be concluded that the sample size used in this study is sufficient and statistically representative (29).

### 2.2 Data collection tools

Data for the study were collected through structured surveys administered directly to participants by the researchers. The survey instrument consisted of a Demographic Information Form and a standardized scale measuring attitudes toward healthy nutrition.

#### 2.2.1 Demographic Information Form

The researchers developed a Demographic Information Form to gather relevant personal and background information about the participants. This form included variables such as age, gender, education level, years of experience in sports, income level, smoking status, and the league in which the athlete competed. This form analyzed potential relationships and differences in nutritional attitudes based on these demographic characteristics.

#### 2.2.2 Attitude scale for healthy nutrition

The Attitude Scale for Healthy Nutrition, developed by Tekkurşun and Cicioglu (30), consists of 21 items across four sub-dimensions and is rated on a five-point Likert scale (one = strongly disagree to five = strongly agree). The sub-dimensions include knowledge about nutrition (items 1–5, α = 0.90), emotion toward nutrition (items 6–11, α = 0.84), Positive Nutrition (items 12–16, α = 0.75), and Malnutrition (items 17–21, α = 0.83). positive items (1–5, 12–16) were scored directly, while negative items (6–11, 17–21) were reverse coded. The total score ranges from 21 to 105, with higher scores indicating more favorable attitudes toward healthy nutrition. Scores are interpreted as follows: 21 = very low, 23–42 = low, 43–63 = moderate, 64–84 = high, and 85–105 = ideally high nutritional attitude. In the current study, the overall Cronbach's alpha reliability coefficient was found to be 0.966, demonstrating excellent internal consistency, and the reliability coefficients for the sub-dimensions remained consistent with those reported in the original scale development study (30).

### 2.3 Analysis of data

The Kolmogorov–Smirnov test was conducted to determine the appropriate tests for data analysis in the study, and the skewness and kurtosis values were examined. According to George and Mallery (31), values within the ±2 range are acceptable for the normality assumption. When this study's skewness and kurtosis values were evaluated, they fell within these acceptable limits. Accordingly, *T*-tests and ANOVA were applied to the data to determine whether there were significant differences in the scores obtained by the athletes across the sub-dimensions. A *post-hoc* (LSD) test was conducted to compare mean differences, as the sample sizes of the comparison groups were unequal. Additionally, partial correlation analyses were performed using IBM SPSS 26 to examine the relationships among the variables.

## 3 Results

**Figure 2:**
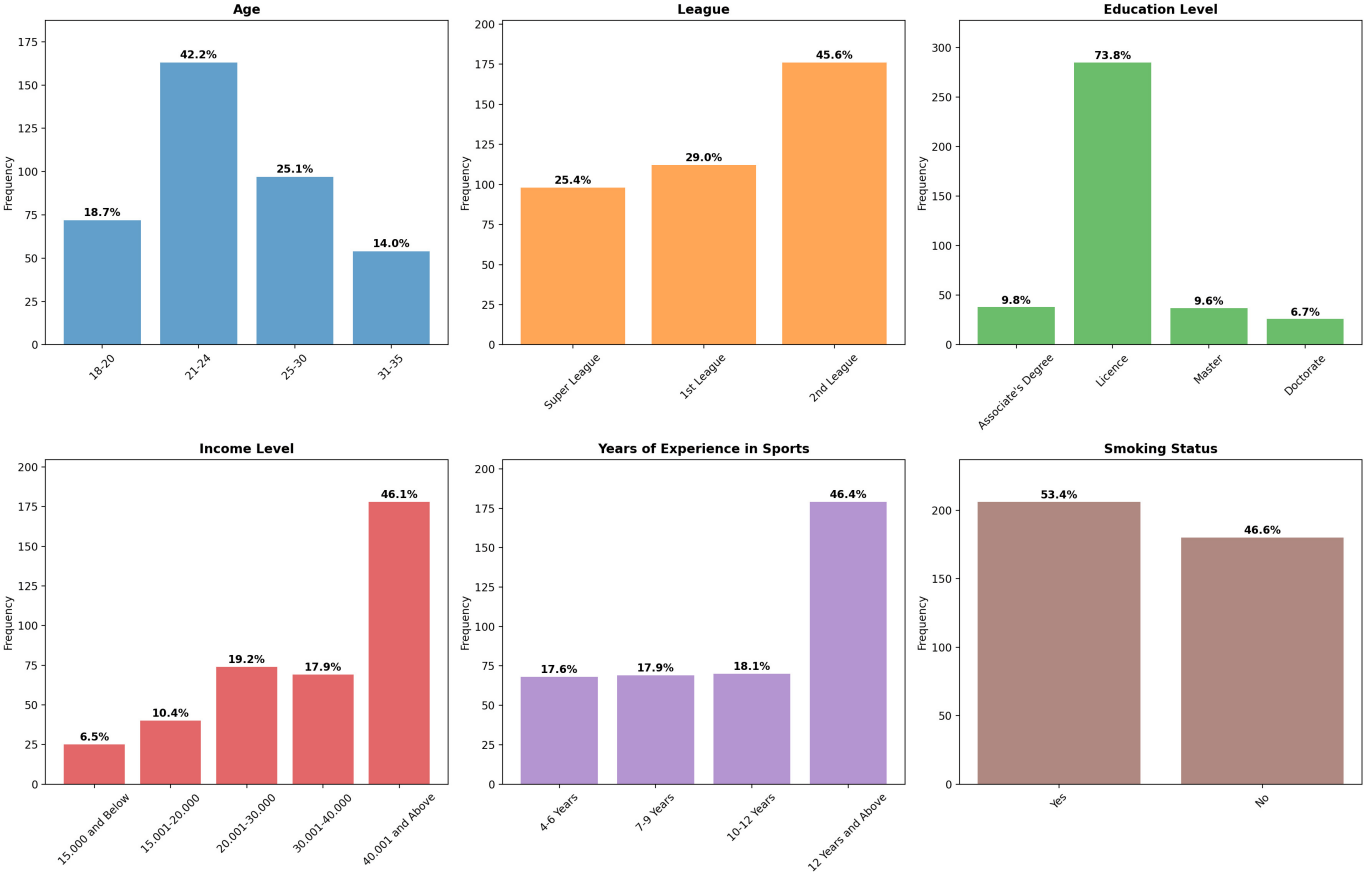
Distribution of demographic and categorical variables.

When we examine the socio-demographic characteristics of the athletes participating in the study (Table 1), we observe 386 female participants. Among the volleyball players, 53.4% smoke, while 46.6% do not. Regarding sports experience, 17.6% of the athletes have 4–6 years, 17.9% have 7–9 years, 18.1% have 10–12 years, and 46.4% have more than 12 years of experience. In terms of income level, 6.5% of the athletes earn 15,000 TL or less, 10.4% earn between 15,001 and 20,000 TL, 19.2% between 20,001 and 30,000 TL, 17.9% between 30,001 and 40,000 TL, and 46.1% earn 40,001 TL or more. As for educational background, 9.8% of the athletes hold an associate degree, 73.8% a bachelor's degree, 9.6% a master's degree, and 6.7% a doctorate. Regarding the leagues they play in, 25.4% of the athletes compete in the Super League, 29% in the 1st League, and 45.6% in the 3rd league. Regarding age distribution, 18.7% of the athletes are between 18 and 20 years old, 42.2% are in the 21–24 age group, 25.1% are in the 25–30 age group, and 14% are in the 31–35 age group. The graphical representation of these findings is presented in Figure 2.

**Table 1:** Descriptive statistics and frequency values of athletes.

**Group**	**Variable**	***F***	**%**
Age	18–20	72	18.7
	21–24	163	42.2
	25–30	97	25.1
	31–35	54	14
League	Super League	98	25.4
	1st League	112	29.0
	2nd League	176	45.6
Education level	Associate's degree	38	9.8
	License	285	73.8
	Master	37	9.6
	Doctorate	26	6.7
Income level	15,000 and below	25	6.5
	15,001–20,000	40	10.4
	20,001–30,000	74	19.2
	30,001–40,000	69	17.9
	40,001 and above	178	46.1
Years of experience in sports	4–6 years	68	17.6
	7–9 years	69	17.9
	10–12 years	70	18.1
	12 years and above	179	46.4
Smoking status	Yes	206	53.4
	No	180	46.6

**Figure 3:**
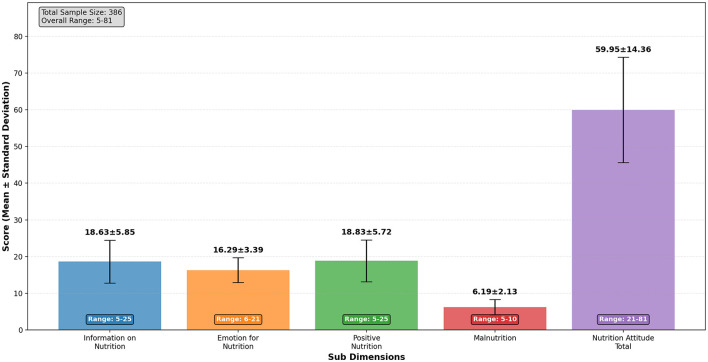
Attitude scale for healthy nutrition- descriptive statistics.

Table 2 presents the descriptive statistics for the sub-dimensions of the Attitude Scale for Healthy Nutrition among professional female volleyball players (*N* = 386). The mean score for the Information on Nutrition sub-dimension was 18.63 (SD = 5.85), indicating moderate nutritional knowledge. The Emotion for Nutrition sub-dimension had a mean score of 16.29 (SD = 3.39), suggesting that participants generally held positive emotional attitudes toward healthy eating. Similarly, the Positive Nutrition sub-dimension scored relatively high (*M* = 18.83, SD = 5.72), further supporting an overall positive orientation toward healthy nutrition. In contrast, the Malnutrition sub-dimension had a lower mean score of 6.19 (SD = 2.13), reflecting a relatively low tendency toward unhealthy eating behaviors. The total score for nutritional attitude averaged 59.95 (SD = 14.36), indicating a moderate to high overall nutritional attitude among the athletes. A graphical representation of these findings is provided in Figure 3.

**Table 2:** Descriptive statistics for sub-dimensions of the attitude scale for healthy nutrition.

	**Sub dimension**	***N***	**Min**.	**Max**.	***X* ±SD**
Attitude scale for healthy nutrition	Information on Nutrition	386	5	25	18.63 ± 5.85
	Emotion for Nutrition	386	6	21	16.29 ± 3.39
	Positive Nutrition	386	5	25	18.83 ± 5.72
	Malnutrition	386	5	10	6.19 ± 2.13
	Nutrition Attitude Total	386	21	81	59.95 ± 14.36

**Figure 4:**
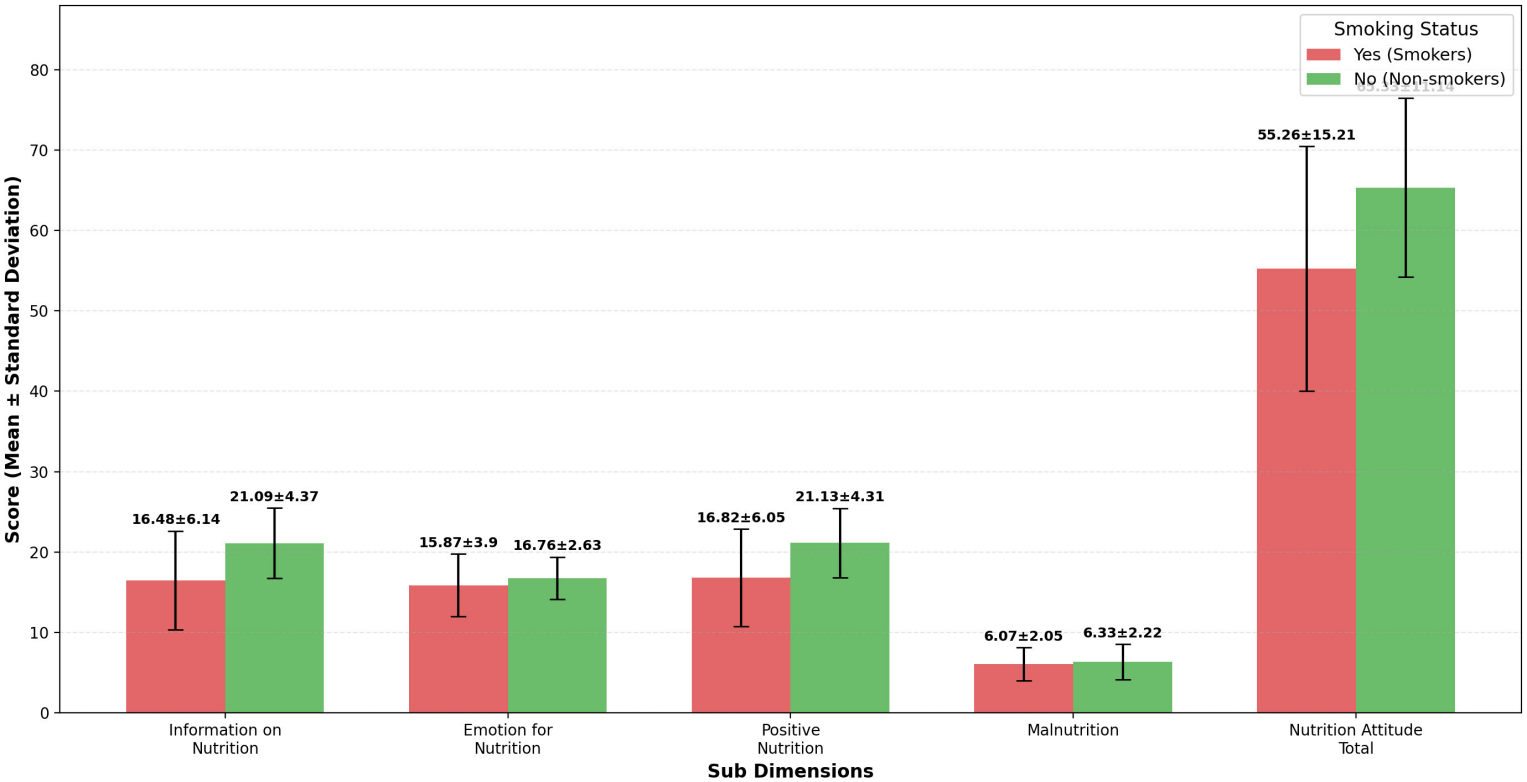
Nutrition attitude sub-dimensions by smoking status (smokers *N* = 206, non-smokers *N* = 180).

When Table 3 is examined, significant differences were found in the total scores of the sub-dimensions of the Attitude Scale for Healthy Nutrition, specifically in Information on Nutrition, Emotion for Nutrition, Positive Nutrition, and overall nutrition attitude, according to the smoking status of the volleyball players (*p* < 0.01). There was a significant difference between smokers and non-smokers in the information on the nutrition dimension (*d* = 0.855), and a slight difference in the emotion for the nutrition dimension (*d* = 0.262). A significant difference was also found in the Positive Nutrition dimension (*d* = 0.814), and a moderate difference in the overall nutrition attitude dimension (*d* = 0.748). No significant difference was observed in the Malnutrition sub-dimension (*p* > 0.01). The findings are illustrated in Figure 4.

**Table 3:** Comparison of volleyball players' attitude scores for healthy nutrition according to the smoking status variable.

**Sub dimension**	**Smoking status**	***N***	***X***	**SD**	***F***	***t***	***p*-Value**	**Cohen's, *d***
Information on Nutrition	Yes	206	16.48	6.14	39.933	−8.568	0.001^**^	0.855
	No	180	21.09	4.37				
Emotion for Nutrition	Yes	206	15.87	3.90	13.395	−2.633	0.009^**^	0.262
	No	180	16.76	2.63				
Positive Nutrition	Yes	206	16.82	6.05	35.011	−8.152	0.001^**^	0.814
	No	180	21.13	4.31				
Malnutrition	Yes	206	6.07	2.05	5.613	−1.202	0.230	
	No	180	6.33	2.22				
Nutrition Attitude Total	Yes	206	55.26	15.21	12.002	−7,482	0.001^**^	0.748
	No	180	65.33	11.14				

^**^p < 0.01, Cohen's d = 0.20 = small effect, 0.50 = medium effect, 0.80 or higher = large effect.

**Figure 5:**
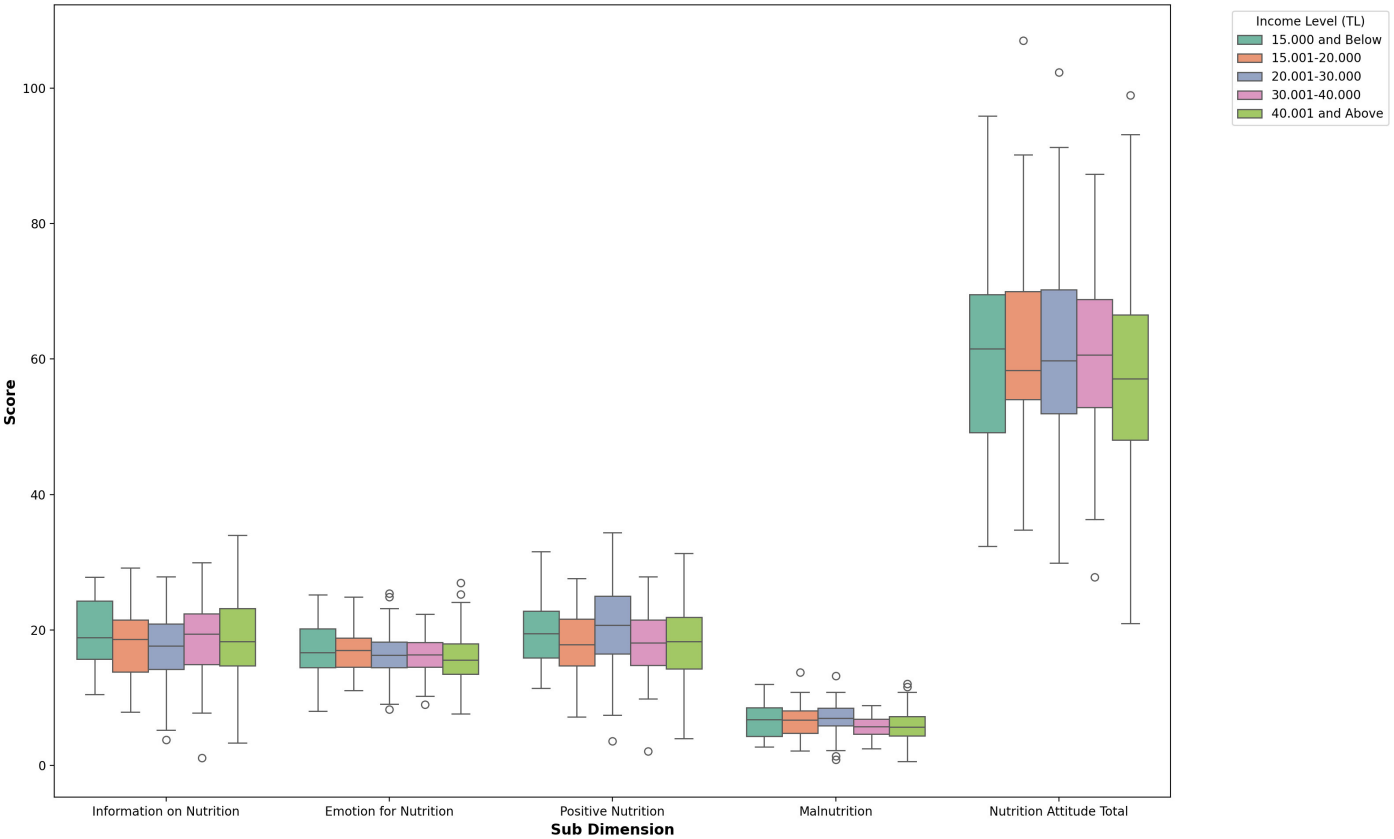
Box plot of nutrition attitude sub-dimensions by income level.

When Table 4 is examined, a significant difference was found only in the *malnutrition* sub-dimension of the Attitude Scale for Healthy Nutrition based on the income levels of the volleyball players. This difference favored players with an income level of 15,000 TL or below. There was a slight difference in Malnutrition scores across several income level comparisons (1–4, 2–4, 3–4, 3–5, and 5–4), with an effect size of *n*^2^ =0.056. No significant differences were observed in the other sub-dimensions. The findings are illustrated in Figure 5.

**Table 4:** Comparison of volleyball players' attitude scores for healthy nutrition according to the income level variable.

**Sub dimension**	**Income level**	***N***	***X***	**SD**	***F***	***p*-Value**	**LSD**	***N* ^2^**
Information on Nutrition	15,000 and below^1^	25	18.56	6.24	0.677	0.608		
	15,001–20,000^2^	40	19.40	6.02				
	20,001–30,000^3^	74	19.30	5.92				
	30,001–40,000^4^	69	17.96	5.71				
	40,001 and above^5^	178	18.46	5.81				
Emotion for Nutrition	15,000 and below^1^	25	16.32	3.88	1.737	0.141		
	15,001–20,000^2^	40	16.73	3.59				
	20,001–30,000^3^	74	17.08	3.03				
	30,001–40,000^4^	69	16.12	3.17				
	40,001 and above^5^	178	15.93	3.48				
Positive Nutrition	15,000 and below^1^	25	18.56	6.24	1.019	0.397		
	15,001–20,000^2^	40	19.40	6.02				
	20,001–30,000^3^	74	19.81	5.62				
	30,001–40,000^4^	69	18.05	5.66				
	40,001 and above^5^	178	18.64	5.65				
Malnutrition	15,000 and below^1^	25	6.80	2.45	5.599	0.001^**^	1 > 4	0.056
	15,001–20,000^2^	40	6.63	2.37			2 > 4	
	20,001–30,000^3^	74	6.96	2.46			3 > 4	
	30,001–40,000^4^	69	5.58	1.61			3 > 5	
	40,001 and above^5^	178	5.94	1.95			5 > 4	
Nutrition Attitude Total	15,000 and below^1^	25	60.24	15.69	1.797	0.129		
	15,001–20,000^2^	40	62.15	14.96				
	20,001–30,000^3^	74	63.15	13.23				
	30,001–40,000^4^	69	57.71	13.68				
	40,001 and above^5^	178	58.97	14.60				

*p* < 0.01^**^, *n*^2^ = 0.01 = small effect, p < 0.06, = medium effect, p < 0.14 large effect. 1 = 15,000 and below, 2 = 15,001–20,000, 3 = 20,001–30,000, 4 = 30,001–40,000, 5 = 40.001 and above.

**Figure 6:**
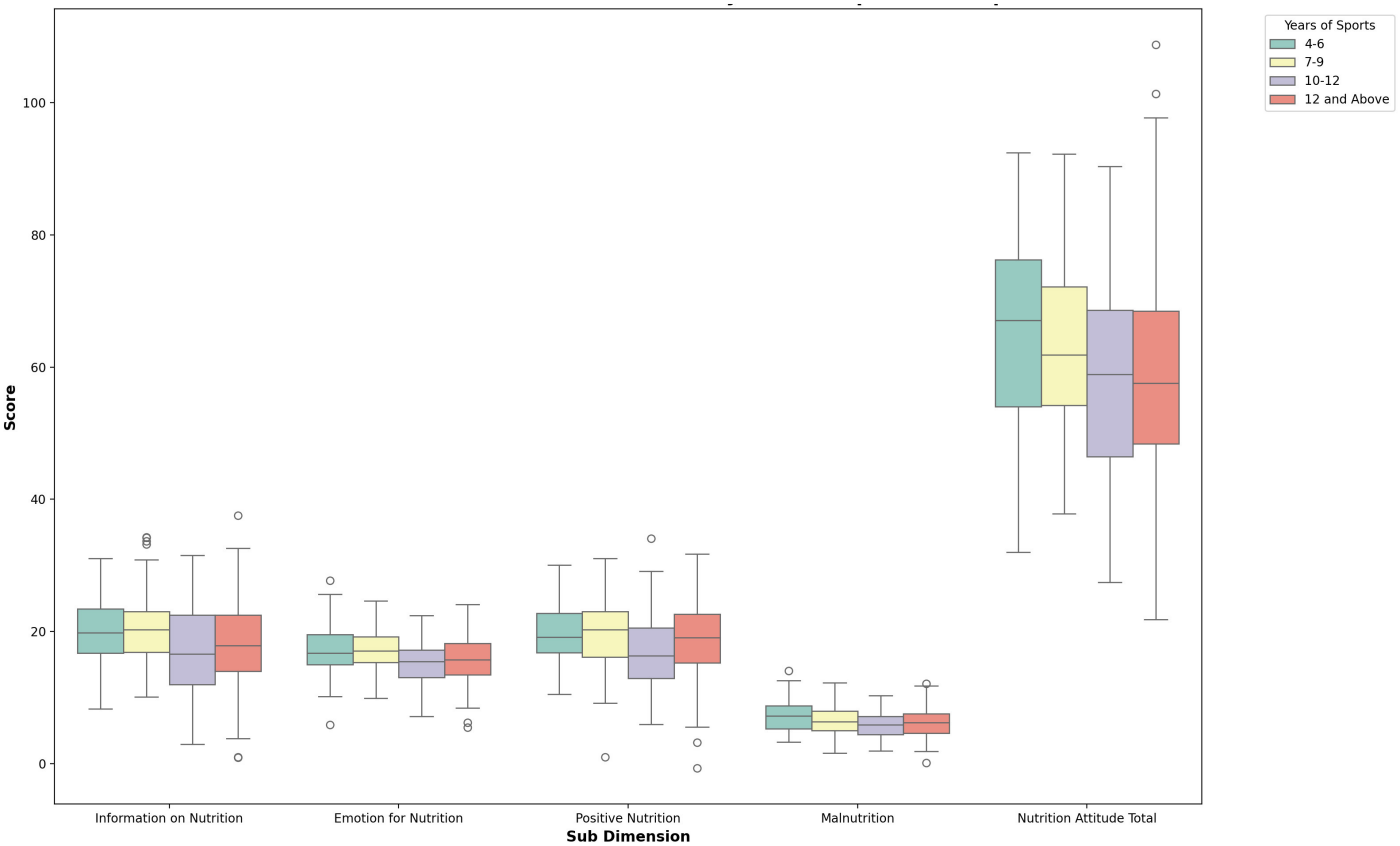
Box plot of nutrition attitude sub-dimensions by years of sports participation.

When Table 5 is examined, differences were found in the sub-dimensions of the Attitude Scale for Healthy Nutrition Emotion for Nutrition, Positive Nutrition, Information on Nutrition, and Overall Nutrition Attitude according to the volleyball players' years of sports experience. A slight difference was observed in Information on Nutrition scores across experience groups (comparisons: 1–3, 1–4, 2–3) with an effect size of η^2^ = 0.032. Similarly, slight differences were found in Emotion for Nutrition (1–3, 1–4, 2–3, 2–4; η^2^ = 0.038), Positive Nutrition (1–3, 1–4, 2–3; η^2^ = 0.032), and overall nutrition attitude scores (1–3, 1–4, 2–3, 2–4; η^2^ = 0.043). No significant difference was found in the Malnutrition sub-dimension. The findings are illustrated in Figure 6.

**Table 5:** Comparison of volleyball players' attitude scores for healthy eating according to years of experience in sports.

**Sub dimension**	**Year of sports**	***N***	***X***	**SD**	***F***	***p*-Value**	**LSD**	***N* ^2^**
Information on Nutrition	4–6^1^	68	20.00	5.88	4.243	0.006^**^	1 > 3 1 > 4 2 > 3	0.032
	7–9^2^	69	19.75	5.07				
	10–12^3^	70	16.96	5.91				
	12 and above^4^	179	18.34	5.95				
Emotion for Nutrition	4–6^1^	68	17.01	3.37	5.006	0.002^**^	1 > 3 1 > 4 2 > 3 2 > 4	0.038
	7–9^2^	69	17.20	2.37				
	10–12^3^	70	15.34	3.25				
	12 and above^4^	179	16.03	3.67				
Positive Nutrition	4–6^1^	68	20.25	5.78	4.152	0.007^**^	1 > 3 1 > 4 2 > 3	0.032
	7–9^2^	69	19.88	4.92				
	10–12^3^	70	17.30	5.75				
	12 and above^4^	179	18.49	5.83				
Malnutrition	4–6^1^	68	6.76	2.41	2.473	0.061		
	7–9^2^	69	6.16	2.13				
	10–12^3^	70	5.80	1.83				
	12 and above^4^	179	6.15	2.11				
Nutrition Attitude Total	4–6^1^	35	66.03	14.03	5.658	0.001^**^	1 > 3 1 > 4 2 > 3 2 > 4	0.043
	7–9^2^	69	63.00	11.42				
	10–12^3^	70	55.40	13.71				
	12 and above^4^	179	59.02	15.17				

*p* < 0.01^**^, *n*^2^ = 0.01 = small effect, *p* < 0.06, = medium effect, *p* < 0.14 large effect, 1 = 4–6, 2 = 7–9, 3 = 10–12, 4 = 12 and above.

**Figure 7:**
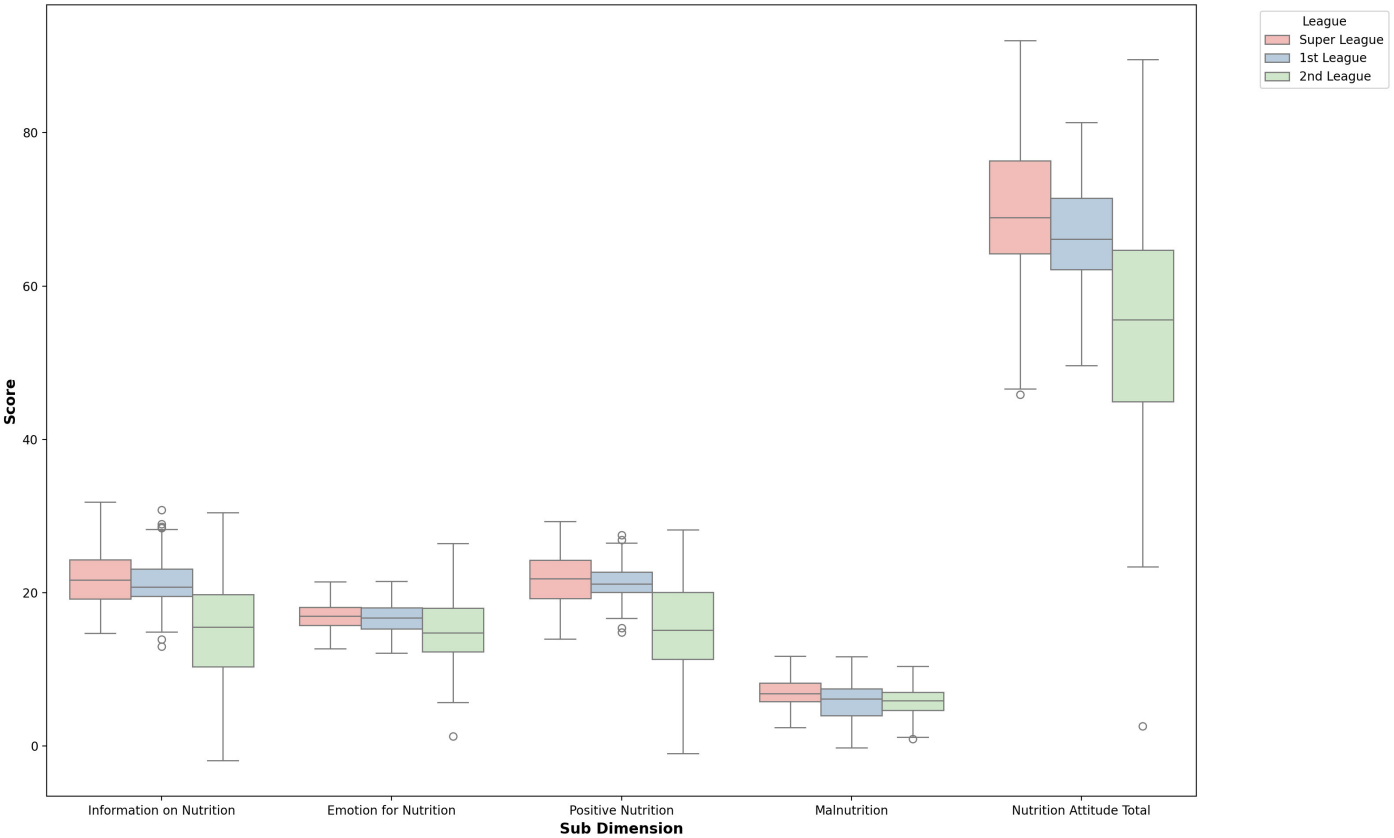
Box plot of nutrition attitude sub-dimensions by league.

When Table 6 is examined, differences were found in the sub-dimensions of the Attitude Scale for Healthy Nutrition Emotion for Nutrition, Positive Nutrition, Information on Nutrition, and Overall Nutrition Attitude according to the leagues in which the volleyball players competed. A significant difference was observed in Information on Nutrition scores between league groups (comparisons: 1–3, 2–3; η^2^ = 0.371), and a moderate difference in Emotion for Nutrition scores (1–3, 2–3; η^2^ = 0.065). Significant differences were also found in *positive nutrition* (1–3, 2–3; η^2^ = 0.345) and overall nutrition attitude scores (1–3, 2–3; η^2^ = 0.312), based on the league level. No significant difference was found in the Malnutrition sub-dimension. The findings are illustrated in Figure 7.

**Table 6:** Comparison of volleyball players' attitude scores for healthy nutrition according to their league.

**Sub dimension**	**League**	***N***	***X***	**SD**	***F***	***p*-Value**	**LSD**	***N* ^2^**
Information on Nutrition	Super League	98	22.14	3.45	112.858	0.001^**^	1 > 3 2 > 3	0.371
	1st League	112	21.67	3.41				
	2nd League	176	14.75	6.10				
Emotion for Nutrition	Super League	98	17.27	2.02	13.299	001^**^	1 > 3 2 > 3	0.065
	1st League	112	16.90	1.93				
	2nd League	176	15.36	4.37				
Positive Nutrition	Super League	98	22.15	3.43	100.990	0.001^**^	1 > 3 2 > 3	0.345
	1st League	112	21.69	2.39				
	2nd League	176	15.17	6.09				
Malnutrition	Super League	98	6.53	2.32	2.474	0.086		
	1st League	112	6.29	2.20				
	2nd League	176	5.95	1.96				
Nutrition Attitude Total	Super League	98	68.09	8.31	86.806	0.001^**^	1 > 3 2 > 3	0.312
	1st League	112	66.55	6.99				
	2nd League	176	51.23	15.58				

*p* < 0.01^**^, *n*^2^ = 0.01 = small effect, *p* < 0.06, = medium effect, *p* < 0.14 large effect. 1 = Super League, 2 = 1st League, 3 = 2nd League.

**Figure 8:**
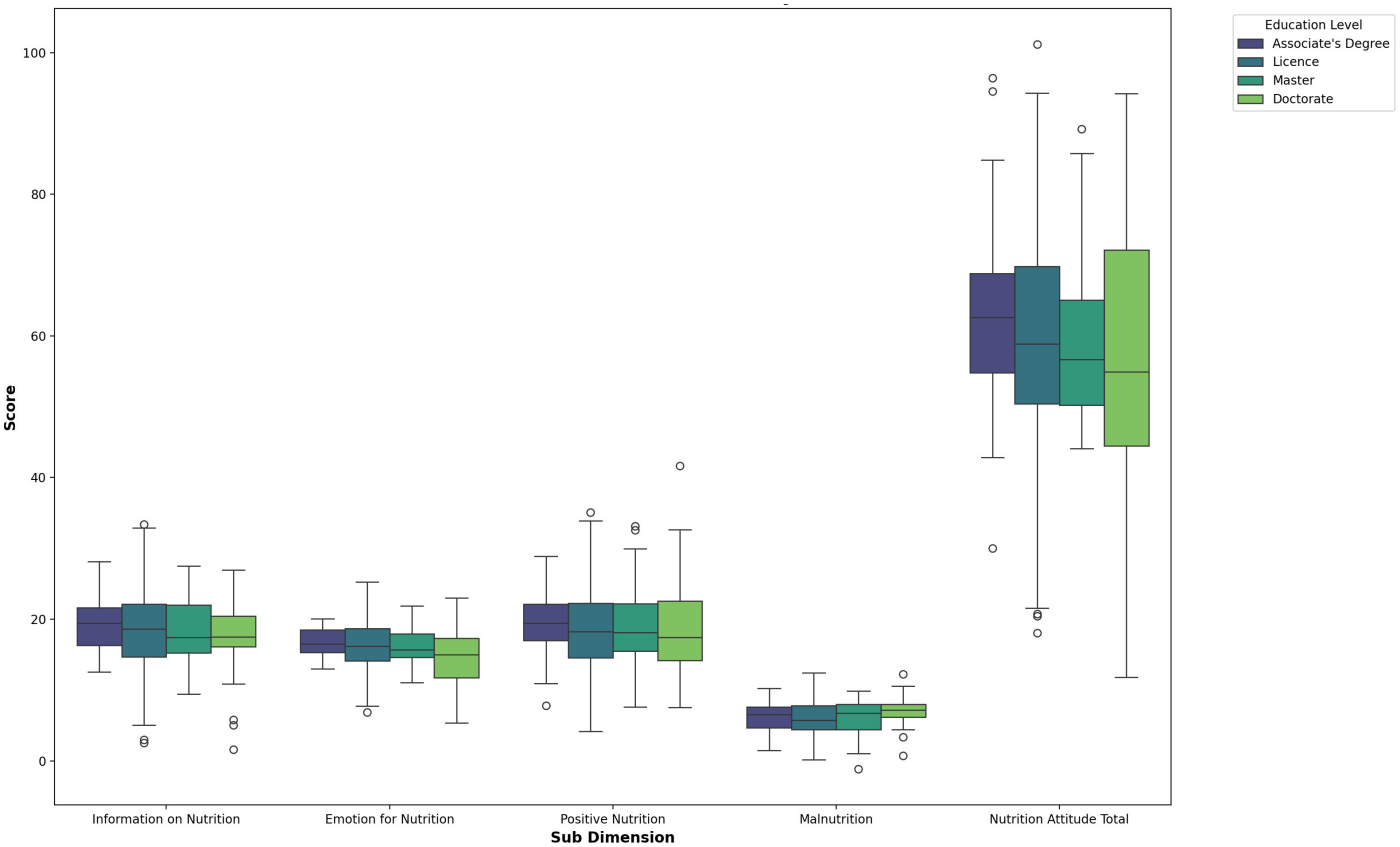
Box plot of nutrition attitude sub-dimensions by education level.

When Table 7 is examined, no significant differences were found in the sub-dimensions of the Attitude Scale for Healthy Nutrition based on the education level of the volleyball players. The corresponding graphical representation is shown in Figure 8.

**Table 7:** Comparison of volleyball players' attitude scores for healthy nutrition according to the education level variable.

**Sub dimension**	**Education level**	***N***	***X***	**SD**	***F***	***p*-Value**	**LSD**	***N* ^2^**
Information on Nutrition	Associate's degree	38	19.21	4.73	0.995	0.395		
	License	285	18.75	5.94				
	Master	37	18.38	5.47				
	Doctorate	26	16.85	6.80				
Emotion for Nutrition	Associate's degree	38	16.63	2.38	0.813	0.487		
	License	285	16.32	3.47				
	Master	37	16.38	2.51				
	Doctorate	26	15.35	4.67				
Positive Nutrition	Associate's degree	38	19.58	4.71	1.129	0.337		
	License	285	18.92	5.82				
	Master	37	18.73	5.14				
	Doctorate	26	17.00	6.71				
Malnutrition	Associate's degree	38	5.82	1.84	2.071	0.104		
	License	285	6.18	2.12				
	Master	37	6.08	2.09				
	Doctorate	26	7.12	2.52				
Nutrition Attitude Total	Associate's degree	38	61.24	11.25	0.689	0.559		
	License	285	60.17	14.56				
	Master	37	59.57	12.30				
	Doctorate	26	56.30	18.59				

A partial correlation analysis was conducted to further explore the relationships between the sub-dimensions while controlling for the league variable. The results are presented in Table 8.

**Table 8:** The relationship between the sub-dimensions of attitudes for healthy nutrition when the league level at which volleyball players play is controlled.

	**Sub dimension**	**1**	**2**	**3**	**4**	**5**
League	Information on Nutrition	*r*	1				
		*p*					
	Emotion for Nutrition	*r*	0.66				
		*p*	0.00^**^				
	Positive Nutrition	*r*	0.96	0.69			
		*p*	0.00^**^	0.00^**^			
	Malnutrition	*r*	−0.08	0.35	−0.09		
		*p*	0.101	0.00^**^	0.08		
	Nutrition Attitude Total	*r*	0.93	0.86	0.94	0.20	1
		*p*	0.00^**^	0.00^**^	0.00^**^	0.00^**^	

^**^p < 0.01, N = 386. 1 = Information on Nutrition; 2 = Emotion for Nutrition; 3 = Positive Nutrition; 4 = Malnutrition; 5 = Nutrition Attitude Total.

Table 8 presents the Fisher *Z* scores for the correlations among the sub-dimensions of the Attitude Scale for Healthy Nutrition: The correlation between Information on Nutrition and Emotion for Nutrition (*r* = 0.66, *p* < 0.001) yielded a *Z* score of 0.7928. The correlation between Information on Nutrition and Positive Nutrition (*r* = 0.96, *p* < 0.001) yielded a *Z* score 1.9459. The correlation between Information on Nutrition and Malnutrition (*r* = −0.08, *p* = 0.10) yielded a *Z* score 0.0802. The correlation between Information on Nutrition and Total Nutrition Attitude (*r* = 0.93, *p* < 0.001) yielded a *Z* score of 1.6584.

The correlation between Emotion for Nutrition and Positive Nutrition (*r* = 0.69, *p* < 0.001) yielded a *Z* score of 0.8480. The correlation between Emotion for Nutrition and Malnutrition (*r* = 0.35, *p* < 0.001) yielded a *Z* score of 0.3654. The correlation between Emotion for Nutrition and Total Nutrition Attitude (*r* = 0.855, *p* < 0.001) yielded a *Z* score of 1.2562.

**Figure 9:**
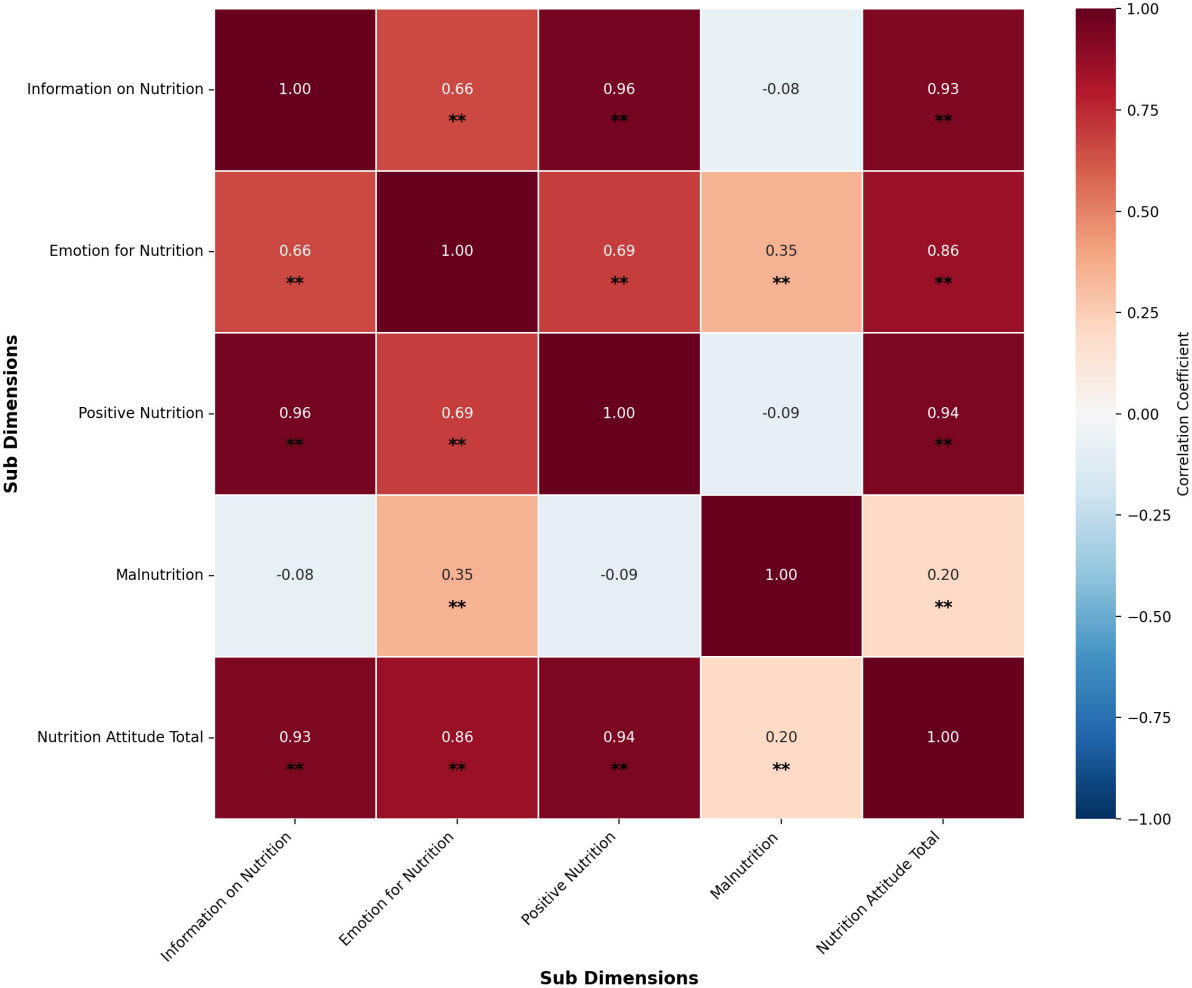
Correlation matrix of nutrition attitude sub-dimensions. *p < 0.01.

The correlation between Positive Nutrition and Malnutrition (*r* = −0.09, *p* = 0.08) yielded a *Z* score of 0.0902. The correlation between Positive Nutrition and Total Nutrition Attitude (*r* = 0.94, *p* < 0.001) yielded a *Z* score of 1.7380. The correlation between Malnutrition and Total Nutrition Attitude (*r* = 0.20, *p* < 0.001) yielded a *Z* score of 0.2027. *Z* scores indicate the strength and direction of the relationships between variables after transforming correlation coefficients. The results are visualized in Figure 9.

## 4 Discussion

This study uses scale data to evaluate female volleyball players' healthy nutritional attitudes and behaviors according to various variables and to reveal the relationships among these factors. The findings revealed significant differences in the sub-dimensions of the Attitude Scale for Healthy Nutrition Emotion for Nutrition, Positive Nutrition, Information on Nutrition, and Overall Nutrition Attitude based on the participants' smoking status. These differences favored non-smoking participants. No significant difference was observed in the Malnutrition sub-dimension. This result contributes to the literature on the negative impact of smoking on healthy eating attitudes. It has been shown that individuals who smoke or consume alcohol tend to develop negative attitudes toward healthy nutrition. In contrast, those who regularly engage in sports adopt more positive attitudes toward exercise and nutrition (32, 33). These findings are supported by previous research.

In their study, Göral and Yildirim (34) reported that scores in the sub-dimensions of Emotion for Nutrition, Positive Nutrition, Malnutrition, and Overall Attitude toward Healthy Nutrition varied depending on academic department. Unlike our study, the sub-dimensions were compared according to the academic department in the literature.

Regarding income levels, the present study found a significant difference only in the Malnutrition sub-dimension. This difference favored players with an income level of 20,001–30,000 TL. No significant differences were found in the other sub-dimensions. This finding may reflect the impact of rising food prices on individuals' nutritional behaviors. During pre-competition periods, athletes who cannot meet their dietary needs may fail to achieve optimal performance. An ideal athletic diet helps maintain optimal body weight, includes the right combination of nutrients, and should be considered a lifestyle (35). Specific nutritional strategies are essential given the demanding and extended nature of professional athletes' training and competition schedules. For example, 100–1,000 mg/day of vitamin C is recommended to aid recovery (36). The liver replenishes glycogen stores 2–3 h before activity, so pre-event meals should contain easily digestible, high-carbohydrate foods. These meals should be low in fat (15%−25% of energy) and fiber, moderate in protein, and composed of familiar foods to reduce gastrointestinal discomfort (37). Post-exercise nutrition should focus on rehydration, replenishing glycogen stores, and synthesizing muscle protein. Foods and fluids consumed after exercise should help restore electrolyte balance and support recovery. Carbohydrates should be combined with adequate protein to facilitate muscle repair, prioritizing fluid intake (37). The literature strongly supports the importance of proper nutrition before, during, and after competition to maintain athletic performance.

The study also found differences in the sub-dimensions Emotion for Nutrition, Positive Nutrition, Information on Nutrition, and Overall Nutrition Attitude based on participants' years in sports. No significant differences were found in the Malnutrition sub-dimension. However, the highest scores in Malnutrition were observed among individuals with 4–6 years of sports experience. This indicates that those with less experience in sports tend to have higher Malnutrition levels than those with more years of experience. Other demographic and lifestyle variables may also contribute to this trend. This finding suggests that sports experience alone is not sufficient to foster healthy eating habits. After years of strict discipline, more experienced athletes may sometimes become complacent or more flexible with their diets. It has been found that individuals who have a positive attitude toward sports are more likely to participate actively and to develop healthy nutritional behaviors (38, 39). Differences between the current findings and those in the literature may be attributed to variations in sample groups and demographic factors.

The present study's findings revealed significant differences in the Emotion for Nutrition, Positive Nutrition, Information on Nutrition, and Nutrition Attitude Total sub-dimensions of the Attitude Scale for Healthy Nutrition based on the leagues in which the volleyball players competed. In particular, Super League athletes' scores for Information on Nutrition were highest, and these scores decreased as the league level declined. This suggests that the more professionally an athlete competes, the higher their nutritional knowledge. Similarly, the Emotion for Nutrition and Positive Nutrition sub-dimensions also favored Super League athletes, with scores decreasing in lower leagues. This implies that the level of professionalism in volleyball is positively associated with healthier emotional and behavioral attitudes toward nutrition. Overall Nutrition Attitude Total scores followed the same trend, supporting the idea that elite athletes adopt more favorable nutrition attitudes. However, no significant difference was found in the Malnutrition sub-dimension across league levels. This could be attributed to standardized nutrition practices among professional athletes in Türkiye, along with similar life routines, body compositions, age distributions, and metabolic needs. Although the Malnutrition subdimension did not demonstrate statistically significant differences across most demographic variables, this uniformity may reflect deeper structural or cultural factors that warrant further discussion. One possible explanation is the relatively homogeneous nutritional awareness and access among athletes regardless of age, gender, or education level, particularly within Turkey's institutional or club-based sports environments. In such contexts, dietary provisions or nutritional guidance may be standardized, reducing group variability. Moreover, socioeconomic status, which often influences nutritional quality, may have been leveled out among participants through access to similar athletic resources. Who observed that organized sports environments can buffer against typical socioeconomic disparities in nutrition. Additionally, the cultural norms around body image and weight control in competitive sports may contribute to a generalized underreporting or normalization of inadequate dietary habits, thereby diminishing perceived differences. Thus, while the lack of variation might initially seem unremarkable, it may reflect a shared vulnerability to nutritional insufficiencies that transcend demographic lines.

When examining the effect of educational attainment on healthy nutrition attitudes, no statistically significant differences were observed across any sub-dimensions. Although participants with associate degrees had slightly higher average scores across all sub-dimensions, this difference was not statistically significant. This finding suggests that general educational level alone does not necessarily influence healthy eating attitudes, particularly without specific nutrition education. Cultural eating habits and other demographic factors may also affect an athlete's nutrition-related behaviors, even professionally. These findings highlight the importance of targeted, subject-specific training in promoting healthy nutritional attitudes. Previous studies align with some of these findings.

Bakhtiar et al. (40) observed variations in athletes' nutritional knowledge based on age, gender, academic field, and duration of sports participation. The differences reported in the relevant literature based on the duration of sports participation are consistent with the findings of our study. Turner et al. (41) found that while gender did not significantly impact nutritional knowledge, age and world ranking had a weak positive effect. The literature has compared different demographic characteristics with those in our study.

When controlling for the league variable, partial correlation analysis revealed a positive and statistically significant relationship between Information on Nutrition and Emotion for Nutrition. This indicates that, at similar league levels, greater nutritional knowledge is associated with more positive emotions toward nutrition. A strong positive correlation was also found between Information on Nutrition and Positive Nutrition, emphasizing that enhanced nutritional awareness contributes to healthier dietary behaviors. No significant relationship was found between Information on Nutrition and Malnutrition, suggesting that increased knowledge alone may not directly reduce unhealthy eating behaviors. On the other hand, a moderate positive correlation emerged between Emotion for Nutrition and Malnutrition, indicating that more emotional involvement in nutrition may sometimes correspond with increased attention to healthy and unhealthy eating behaviors. No significant relationship was found between Positive Nutrition and Malnutrition, implying different factors may independently influence these constructs. The Fisher *Z*-test results confirmed the direction and strength of the relationships between Information on Nutrition, Emotion for Nutrition, Positive Nutrition, and Nutrition Attitude Total, validating the internal consistency of the findings.

## 5 Conclusions

In summary, the study demonstrated statistically significant differences in the Information on Nutrition, Emotion for Nutrition, Positive Nutrition, and Nutrition Attitude Total sub-dimensions according to the participants' smoking status, sports experience, and league levels (*p* < 0.05), with no significant difference observed in the Malnutrition sub-dimension (*p* > 0.05). Additionally, while income level significantly affected Malnutrition scores (*p* < 0.05), it did not impact other sub-dimensions. No significant differences were found in any dimension based on education level (*p* > 0.05). A strong correlation was found between nutritional attitudes and league level, particularly regarding nutritional knowledge, emotion, and positive attitudes (*p* < 0.05). A significant relationship was also found between Malnutrition and Emotion for Nutrition (*p* < 0.05), although no other variable showed a significant correlation with Malnutrition. These results indicate that professional female volleyball players' nutritional attitudes differ significantly based on key demographic characteristics. Consequently, the findings highlight the critical need to shift away from traditional seminar-based nutrition education and toward more personalized, athlete-specific programs. Future interventions should include practical workshops, tailored meal plans, performance-based nutrition strategies, and interactive learning components to translate nutritional awareness into lasting behavior change. Furthermore, establishing a dynamic, expert-supervised, and trackable nutrition education system would enhance athletes' health and performance outcomes. These findings represent valuable suggestions for sports clubs and governing bodies and a compelling call to action for implementing more effective and targeted nutrition education programs.

## 6 Limitations of the study

This study was limited to female athletes competing in the Turkish Women's Volleyball Super League, 1st League, and 2nd League.

Only athletes who had been licensed volleyball players for at least 4 years and actively played in these leagues were included.

Participants were required to be over the age of 18.

Data collection was restricted to a demographic information form and the Attitude Scale for Healthy Nutrition.

The study examined specific demographic variables, including years of experience in sports, income level, education level, league of participation, smoking status, and age. Other potentially influential factors, such as dietary environment, cultural background, and access to nutrition education, were not assessed and thus represent areas outside the scope of this study.

## 7 Future research

Future studies could adopt an experimental or longitudinal design to evaluate changes in nutrition attitudes before and after structured nutrition education interventions among volleyball players. Such studies may help to determine the direct impact of tailored nutrition training programs on athletes' attitudes and behaviors.

Further research could explore the relationship between nutrition attitudes and athletes' annual league performance, allowing for performance-based evaluations across different teams and divisions.

Comparative studies involving athletes from other sports disciplines could provide broader insights into how sport-specific factors influence nutritional attitudes.

Additionally, demographic and personal variables such as age, weight, height, regional dietary practices, and cultural eating habits may be integrated into future analyses to understand better the factors influencing athletes' nutritional attitudes.

## 8 Suggestions

Providing structured and evidence-based nutrition education to volleyball players has the potential to positively influence dietary behaviors and prevent performance losses due to poor nutrition. Special attention should be directed toward 1st and 2nd League athletes, who may benefit from targeted interventions to elevate their nutritional knowledge and attitudes to the level observed in Super League athletes.

Coaches, in collaboration with certified sports nutritionists, should consider developing individualized meal plans tailored to each athlete's physiological needs, training load, and competitive schedule. These plans can support the establishment of consistent and health-promoting eating routines within teams.

Moreover, regular monitoring and strategic regulation of key demographic and behavioral variables such as smoking status, sports experience, league level, income, education, and dietary habits can enhance players' nutritional wellbeing and athletic performance. Integrating these practices into training regimens can contribute to more sustainable performance outcomes and improved overall athlete health.

The findings of this study provide several actionable insights for coaches, sports nutritionists, and sports federations. Firstly, the identified nutritional risk patterns can guide the development of tailored intervention programs, particularly for athletes competing in lower-tier leagues who may lack regular access to professional dietary support.

Coaches can utilize these findings to incorporate basic nutrition education into training routines, raising athletes' awareness of performance-related dietary needs. Similarly, sports nutritionists can design low-cost, accessible meal planning strategies that align with the specific demands of different sports and competition levels.

Sport federations are encouraged to allocate resources toward periodic nutritional assessments and workshops, especially targeting demographic groups or disciplines with high risk levels.

By implementing these measures, stakeholders can enhance overall athlete wellbeing, reduce performance variability due to poor nutrition, and promote long-term health in competitive environments.

## Data Availability

The original contributions presented in the study are included in the article/supplementary material, further inquiries can be directed to the corresponding author.
